# Collagen Peptide in a Combinatorial Treatment with *Lactobacillus rhamnosus* Inhibits the Cariogenic Properties of *Streptococcus mutans*: An In Vitro Study

**DOI:** 10.3390/ijms23031860

**Published:** 2022-02-07

**Authors:** Hee-Young Jung, Jian-Na Cai, Sung Chul Yoo, Seon-Hwa Kim, Jae-Gyu Jeon, Dongyeop Kim

**Affiliations:** 1 Department of Preventive Dentistry, School of Dentistry, Jeonbuk National University, Jeonju 54896, Korea; ghgh7090@jbnu.ac.kr (H.-Y.J.); caijianna1110@163.com (J.-N.C.); dentjjk@jbnu.ac.kr (J.-G.J.); 2 Vixxol Corporation, Gunpo 15807, Korea; scyoo@vixxol.com (S.C.Y.); seonhkim@vixxol.com (S.-H.K.); 3 Institute of Medical Information Convergence Research, Jeonbuk National University, Jeonju 54896, Korea

**Keywords:** synbiotics, probiotics, prebiotics, cariogenic biofilms, metabolic interference, acidogenicity, aciduricity

## Abstract

Dental caries is caused by the formation of cariogenic biofilm, leading to localized areas of enamel demineralization. *Streptococcus mutans*, a cariogenic pathogen, has long been considered as a microbial etiology of dental caries. We hypothesized that an antagonistic approach using a prebiotic collagen peptide in combination with probiotic *Lactobacillus rhamnosus* would modulate the virulence of this cariogenic biofilm. In vitro *S. mutans* biofilms were formed on saliva-coated hydroxyapatite discs, and the inhibitory effect of a combination of *L. rhamnosus* and collagen peptide on *S. mutans* biofilms were evaluated using microbiological, biochemical, confocal imaging, and transcriptomic analyses. The combination of *L. rhamnosus* with collagen peptide altered acid production by *S. mutans*, significantly increasing culture pH at an early stage of biofilm formation. Moreover, the 3D architecture of the *S. mutans* biofilm was greatly compromised when it was in the presence of *L. rhamnosus* with collagen peptide, resulting in a significant reduction in exopolysaccharide with unstructured and mixed bacterial organization. The presence of *L. rhamnosus* with collagen peptide modulated the virulence potential of *S. mutans* via down-regulation of *eno*, *ldh*, and *atpD* corresponding to acid production and proton transportation, whereas *aguD* associated with alkali production was up-regulated. Gly-Pro-Hyp, a common tripeptide unit of collagen, consistently modulated the cariogenic potential of *S. mutans* by inhibiting acid production, similar to the bioactivity of a collagen peptide. It also enhanced the relative abundance of commensal streptococci (*S. oralis*) in a mixed-species biofilm by inhibiting *S. mutans* colonization and dome-like microcolony formation. This work demonstrates that food-derived synbiotics may offer a useful means of disrupting cariogenic communities and maintaining microbial homeostasis.

## 1. Introduction

Dental caries is one of the most prevalent biofilm and diet-dependent oral diseases worldwide, resulting in annual expenditures of over USD 40 billion and afflicting mostly underprivileged persons [1,2]. In this disease, a cariogenic pathogen, *Streptococcus mutans,* is often found along with other microorganisms [3,4,5]. A sugar-rich diet (i.e., a diet rich in processed foods) promotes *S. mutans* assembly with an exopolysaccharide (EPS)-rich matrix via glucosyltransferase (Gtf) exoenzymes and acidifies the microenvironment through constant sugar catabolism [6]. An EPS-rich and acidic microenvironment is a key virulence factor that acts as a three-dimensional (3D) scaffold and protection barrier to diffusion, enhancing the cariogenic potential of an *S. mutans*-dominated biofilm. In particular, a bacteria-derived EPS-matrix enhances drug resistance, as adhered microbes are enmeshed within a shield that protects against antibiotics [7,8].

Oral probiotics have been considered an alternative approach to the disruption of cariogenic biofilms [9,10]. The administration of oral probiotics to cariogenic microbiota helps maintain a healthy microbial community by suppressing bacterial colonization and the virulence mechanism of *S. mutans*. However, current studies focused on the preventative potential of conventional probiotics (which have mostly examined *Lactobacillus* used in treating gastrointestinal disorders) on dental caries have shown inconsistent results [11,12]. Even when the *Lactobacillus rhamnosus* strain GG is shown to have the potential for modulating the virulence of *S. mutans*-mediated biofilms [12,13], it may have a limited ability to prevent colonization of *S. mutans* on the tooth surface [14]. Since lactobacilli are often considered late colonizers of the pellicle, probiotics alone are insufficient to prevent cavities [10,11]. To accelerate the use of conventional probiotics, which have well-characterized and well-documented health benefits, an adjuvant for that promotes the colonization of probiotics while modulating the virulence factor of the pathogen is necessary [15,16].

Prebiotics impact the functional potential of bacterial cells [15,16]. Therefore, probiotics are in a combination of prebiotics (referred to as synbiotics), that synergically modulate the virulence of *S. mutans* and, in turn, inhibit cariogenic biofilm formation [17]. An enhanced understanding of the preventive implications of synbiotics may allow for improved antibiofilm strategies that overcome the limitations of current modalities by applying antibiotics. We hypothesized that probiotic *L. rhamnosus* administered in combination with prebiotic collagen peptide (CP) would thwart *S. mutans* outgrowth under cariogenic conditions. To address this, we examined whether *L. rhamnosus* modulated the virulence factors of *S. mutans* in the presence of CP by modifying a cariogenic biofilm structure, while rearranging bacterial organization to cause defective EPS matrix formation. We further investigated whether the introduction of synbiotics promotes the homeostasis of commensal via modifying acidogenic and aciduric properties of *S. mutans*.

Thus, this study aims to investigate the inhibitory effect of CP in a combination with *L. rhamnosus* on cariogenic potential of *S. mutans* using a well-established saliva-coated hydroxyapatite in vitro biofilm model.

## 2. Results

### 2.1. Effects of Prebiotic Collagen Peptide and Probiotic L. rhamnosus on Bacterial Cell Growth

**Figure 1:**
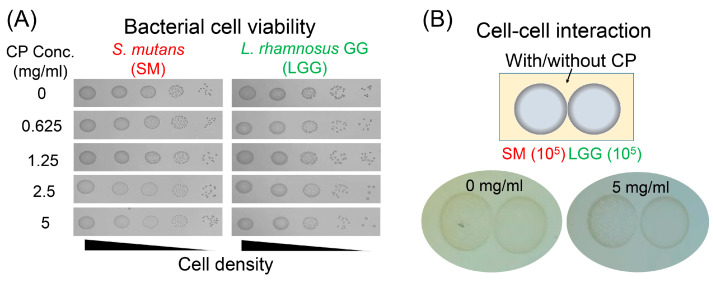
Effects of collagen peptide (CP) on bacterial cell viability and cell–cell interaction between *S. mutans* and *L. rhamnosus*. (**A**) Effects of CP on the cell viability of *S. mutans* and *L. rhamnosus*. CP demonstrated no inhibitory activity on the growth of *S. mutans* and *L. rhamnosus* in brain heart infusion (BHI) agar plate. (**B**) Cell–cell interaction between *S. mutans* and *L. rhamnosus* with or without CP. Where one strain is capable of competing with another, the growth of spotted bacterial macrocolony shows defective patterns by another (left, *S. mutans* (SM); right, *L. rhamnosus* (LGG)). There is no direct growth competition between two bacteria in the BHI agar plate.

The influences of CP on the cell viability of *S. mutans* and *L. rhamnosus* were determined by agar plate assay. To investigate the effect of CP supplementation on bacterial growth, serially diluted bacterial culture (ranged 10^3–7^ CFU/mL) was spotted on a CP-supplemented agar plate (0–5 mg/mL). As shown in Figure 1A, there was no growth-inhibitory activity against either *S. mutans* or *L. rhamnosus*. This is consistent with the idea of prebiotics, according to which the agent does not kill or inhibit the cell growth [15,18]. We further conducted a competition assay to evaluate whether *L. rhamnosus* (ranged 10^5–7^ CFU/mL) inhibits pathogenic *S. mutans* (10^5^ CFU/mL) growth on a CP-supplemented agar plate (Figure 1B). There was no direct inhibition of *S. mutans* by *L. rhamnosus* regardless of the presence or absence of CP.

### 2.2. Effect of Collagen Peptide on Glycolytic Acid Production by S. mutans and L. rhamnosus

**Figure 2:**
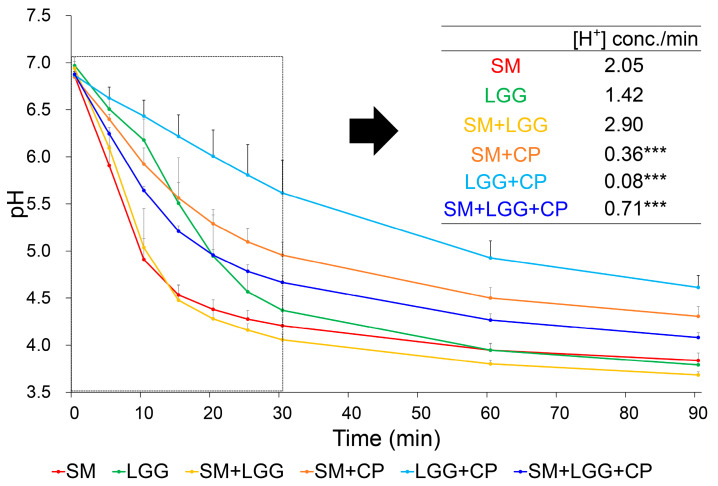
Effects of CP on the glycolytic pH drop by *S. mutans* and *L. rhamnosus* planktonic cells. The initial rate of the pH drop was calculated by hydrogen ion (H^+^) released for 30 min (dashed blot box). The molar concentration of H^+^ in the solution was estimated via the equation: [H^+^] = 10^-pH^. Data represent mean ± standard deviations (SD) (*n* = 3). Values are significantly different from each other (*t*-test for a pairwise comparison, with CP vs. without CP) at *** *p* < 0.001.

A glycolytic pH drop assay was performed to evaluate the acid production ability of planktonic *S. mutans* in coexistence with *L. rhamnosus* in the presence of CP. As shown in Figure 2, over the 90 min experiment period, CP increased the pH of *S. mutans* cell suspension (low aciduricity) regardless of the presence of *L. rhamnosus*. Following CP treatment, the rate of acid production of *S. mutans* was reduced by 82% (*p* < 0.001). In parallel with a reduction in [H^+^] concentration of *S. mutans*, CP also inhibited glycolytic pH drop of *L. rhamnosus* itself or together with *S. mutans*, resulting in a 94% and 75% reduction (versus vehicle control (without CP)), respectively (low acidogenicity).

### 2.3. Effect of Collagen Peptide in a Combination of L. rhamnosus on the Biofilm Formation of S. mutans

**Figure 3:**
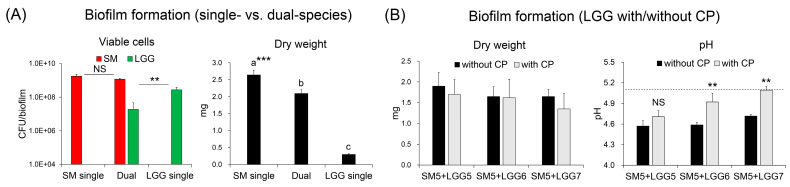
Biofilm formation of *S. mutans* and *L. rhamnosus* single or dual-species bioflim (42 h-old) with or without CP. (**A**) Viable cell counts (colony forming units (CFU)/biofilm) and dry weight of *S. mutans* and *L. rhamnosus* single or dual-species bioflim. The inoculum populations of both *S. mutans* and *L. rhamnosus* were 10^5^ colony-forming units (CFU)/mL. (**B**) Dry weight and culture pH of dual-species biofilm with or without CP. Dot-line indicates the pH value of *S. mutans* (10^5^) and *L. rhamnosus* (10^7^) is above the critical pH of demineralization (pH 5.0). Data represent mean ± SD (n = 4). The data were subjected to analysis of variance (ANOVA) in the Tukey’s HSD test for a multiple comparison or student’s *t*-test for a pairwise comparison (single vs. dual or without CP vs. with CP). Values followed by different letters (a-c in a multiple comparison) are significantly different from each other at ** *p* < 0.01, *** *p* < 0.001. NS, no significance.

In single- and dual-species biofilms (42 h-old), colonized viable cells of *S. mutans* and *L. rhamnosus* were between 10^8^ to 10^9^ CFU/mL, indicating that both were well colonized on the hydroxyapatite (HA) discs. However, the total biomass (dry weight) of *L. rhamnosus* was 10 times less than that of the *S. mutans* biofilm and, moreover, that of the coculture of *S. mutans* and *L. rhamnosus* was significantly reduced without drastic changes to the *S. mutans* cells in the biofilms (Figure 3A). We further tested whether the different inoculums of *L. rhamnosus* (10^5^ to 10^7^ CFU/mL) affected the biomass and culture pH in the presence of CP (5 mg/mL). The results showed that 10^7^ CFU/mL *L. rhamnosus* with CP slightly reduce the dry weight while a culture pH of biofilm was increased to the critical pH point (about pH 5.0) (Figure 3B). In sum, the combination of probiotic *L. rhamnosus* and prebiotic CP exhibited no bactericidal effect on *S. mutans* but did interfere with its metabolic traits (e.g., reduced acid production).

**Figure 4:**
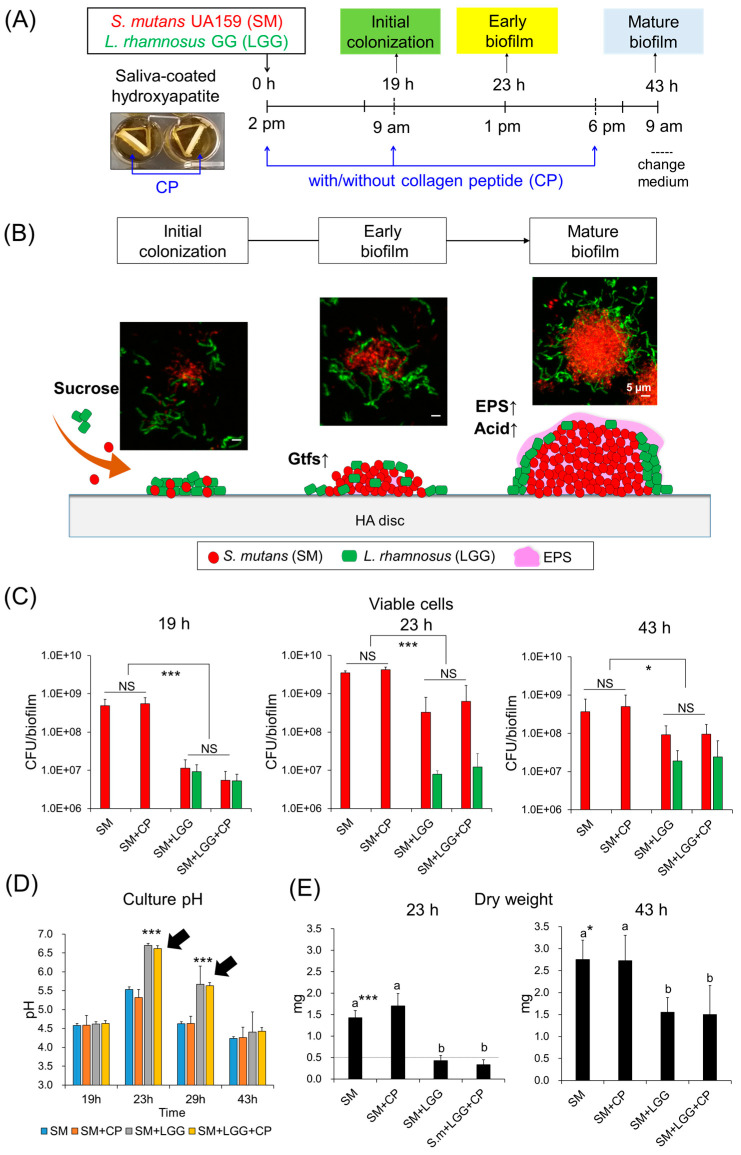
Experiment scheme and microbiological and biochemical analysis of biofilms. (**A**) Experiment design for biofilm formation and CP treatment. The mixed population was inoculated in 2.8 mL of medium containing 1% (*w*/*v*) sucrose with/without CP and incubated at 37 °C and 5% CO_2_. The culture medium containing 1% (*w*/*v*) sucrose was changed twice daily (at 9 am (19 h) and 6 pm (28 h)). (**B**) Schematic diagram of development stages of biofilm. Viable cell counts (**C**), culture pH (**D**) and dry weight (**E**) of *S. mutans* single biofilm or cocultured bioflim with *L. rhamnosus* in the presence or absence of CP. Dot-line indicates the detection limits of dry weight measurement. Data represent mean ± SD (*n* = 4). Values followed by different letters (a, b) are significantly different from each other at * *p* < 0.05, *** *p* < 0.001. NS, no significance.

Next, the combined effect of *L. rhamnosus* and CP on *S. mutans* biofilm development was assessed. Using an established biofilm model (Figure 4A), in which the viable cell, dry weight, culture pH, and 3D architecture of mono- or dual-species biofilms between *S. mutans* and *L. rhamnosus* in the presence or absence of CP were determined at three different times (19, 23, and 43 h). These different time points were corresponded to initial attachment, early-stage, and mature biofilms, reflecting intermixed cell attachment to microcolony formation (Figure 4B). As shown in Figure 4D,E, the presence of *L. rhamnosus* with/without CP resulted in reduction in dry weight, while the culture pH of the biofilm was significantly increased. The viable cell counts were significantly decreased in the presence of *L. rhamnosus* and CP, especially at the stage for the initial colonization (19 h), which showed a 2-log CFU reduction (Figure 4C). In contrast, CP alone did not display any influence on *S. mutans* biofilm formation across the entire experiment period.

Since total biomass reflects the sum of cells and the extracellular matrix, we also investigated the 3D architecture of the biofilm by using bacterial species-specific probes and the EPS labeling technique. This was essential to determining the bacterial cell assembly and EPS distribution in the presence of *L. rhamnosus* and CP. To further determine any inhibitory effects of the combinatorial treatment of *L. rhamnosus* with CP on the alternation of the 3D architecture of the *S. mutans* biofilm, we performed confocal imaging with quantitative computational analysis.

**Figure 5:**
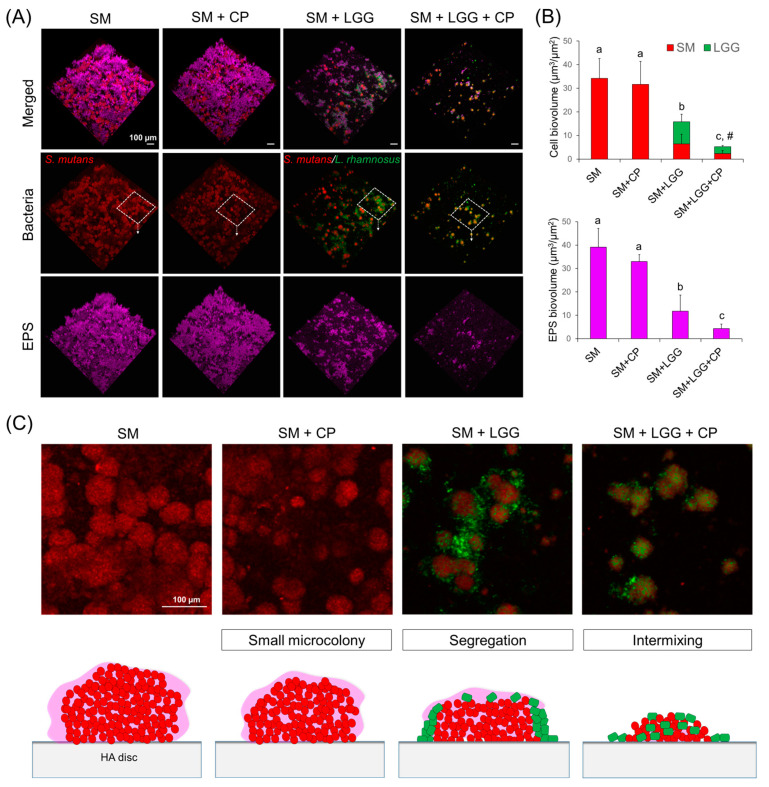
Three-dimensional (3D) architecture and quantitative computational analysis of *S. mutans* biofilm (23 h-old) with or without *L. rhamnosus* and CP. (**A**) Representative confocal images, *S. mutans* and *L. rhamnosus* cells are depicted in red and green, respectively; while the EPS-matrix is depicted in purple. (**B**) Quantitative analysis of bacteria cell and EPS biovolume was performed using COMSTAT. (**C**) Close-up images of selected areas (dashed box) and schematic diagram of biofilm model. Data represent mean ± SD (*n* = 10). Values followed by different letters (a–c) are significantly different from each other (*p* < 0.001). # indicates that cell biovolume of both *S. mutans* and *L. rhamnosus* in the presence of CP (SM + LGG + CP) is significantly different in a pair comparison with vehicle control (SM + LGG). SM, *S. mutans*; LGG, *L. rhamnosus* GG; CP, collagen peptide.

Representative images showed that *L. rhamnosus* combined with CP resulted in a marked impairment of EPS-matrix accumulation and microcolony development, which exhibited sparse amounts of EPS randomly interspersed among bacterial cells without any structural organization, resulting in reduced bacteria–EPS co-localization (Figure 5A). This result was consistent with computational imaging analysis, which showed approximately 9 times less EPS in biofilms treated with *L. rhamnosus* and CP (vs. single *S. mutans* biofilms) (Figure 5B). Furthermore, a close-up image of the selected area revealed that the presence of CP or *L. rhamnosus* partially resulted in small microcolony and loose structure of *S. mutans* clusters, but that a combination of *L. rhamnosus* and CP further inhibited the accumulation of *S. mutans*, resulted in cell clusters intermixed with *L. rhamnosus* (Figure 5C). In sum, the data indicate that a combination of CP and *L. rhamnosus* substantially inhibits initial colonization of *S. mutans* and EPS-matrix formation, thereby reducing the bulk and density of infection.

### 2.4. Effect of Collagen Peptide in a Combination of L. rhamnosus on the Transcriptomic Changes of S. mutans Biofilm

**Figure 6:**
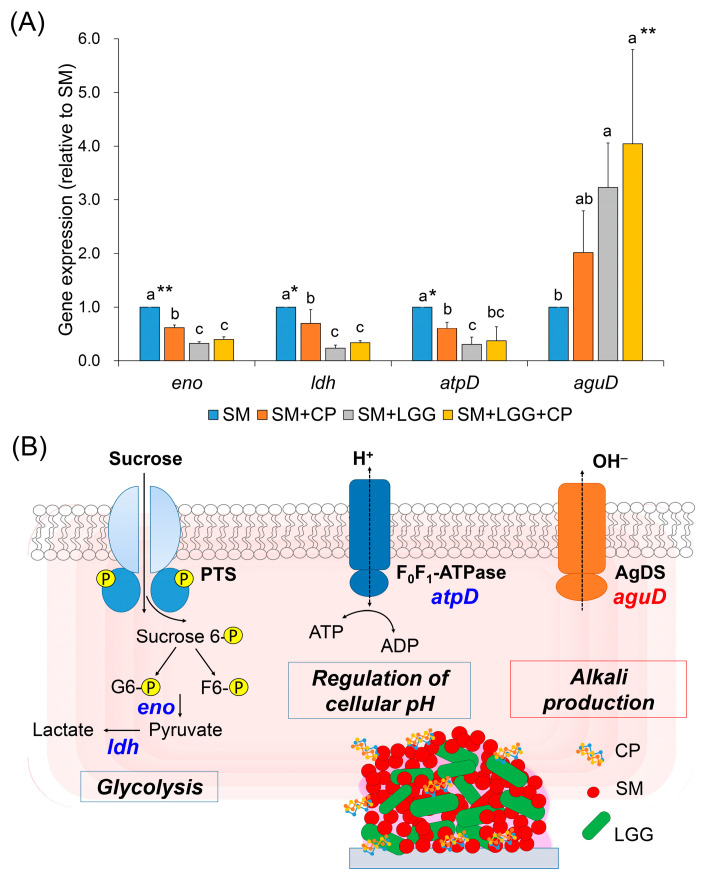
Gene expression of *S. mutans* biofilm with or without *L. rhamnosus* and CP (**A**) and postulated pathways of acid production and acid tolerance in *S. mutans* (**B**). The data reveal that the genes related to acidogenic and aciduric properties of *S. mutans* was inhibited in the presence of *L. rhamnosus* and CP via down-regulation of *eno*, *ldh,* and *atpD* genes (associated with acid production; blue letters), whereas upregulation of *aguD* gene (associated with alkali production; red letters). Data represent mean ± SD (*n* = 4). Values followed by different letters (a–c) are significantly different from each other at * *p* < 0.05, ** *p* < 0.01. SM, *S. mutans*; LGG, *L. rhamnosus* GG; CP, collagen peptide.

To further understand mechanism of action of a combinatorial treatment of *L. rhamnosus* with CP, we examined the potential effects of the combinatorial treatment on the expression of specific *S. mutans* genes using quantitative real-time PCR (qRT-PCR). The genes selected for qRT-PCR were related to a transcription factor of acid production (*eno* and *ldh*) and acid tolerance (*atpD* and *aguD*). The gene expression of *S. mutans* in an early-stage biofilm were analyzed since the combinatorial treatment drastically modified culture pH and altered 3D architecture of biofilms at 23 h. As shown in Figure 6, *L. rhamnosus,* together with CP, repressed the expression of genes associated with glycolysis by *S. mutans*. In addition, the expression of *ldh*, which encodes *S. mutans* lactate dehydrogenase (responsible to acidogenicity) was down-regulated, while *aguD* expression, which is related to acid tolerance, was up-regulated (promoting alkali (e.g., ammonia) production) in the presence of *L. rhamnosus* and CP. This was consistent with results from the glycolytic pH drop assay, in which CP decreased the acid production rate (Figure 2). Since *eno* and *ldh* are involved in the synthesis of pyruvate and lactate, it is possible that the combinatorial treatment is capable of inhibiting acid production. Additionally, the *atpD* gene corresponding to proton transportation through F-ATPase was also repressed. However, *aguD* is associated with agmatine catabolism via the agmatine deiminase system (AgDS), a pathway that utilizes an agmatine deiminase to hydrolyze agmatine into putrescine, during which concomitantly released ammonia alters the acidic environment [19]. Thus, upregulation of *aguD* expression by combinatorial treatment is also consistent with an increase in culture pH (up to pH 6.6) (Figure 4D). In sum, the data suggest that *L. rhamnosus* with CP can modulate the cariogenic potential of *S. mutans* by regulating acidogenicity- and acid tolerance-related gene expression.

### 2.5. Effects of Glycine-Proline-Hydroxyproline (Gly-Pro-Hyp) and the Presence of L. rhamnosus on the Acid Production Ability of S. mutans

Since collagen is composed of various peptides and amino acids, we further assessed the potential effect of a collagen tripeptide rich in Gly-Pro-Hyp, and glycine (Gly), one of most abundant amino acids in CP, on glycolytic acid production by *S. mutans* and *L. rhamnosus*. As shown in Table 1, treatment with Gly-Pro-Hyp or Gly significantly decreased the extracelluar [H^+^] concentration of the bacterial culture, which showed a similar inhibition activity to CP. Furthermore, Gly-Pro-Hyp exhibited the lowest [H^+^] production rate (0.04) compared to vehicle control (expressed as 1.00).

### 2.6. Gly-Pro-Hyp Treatment Prevented S. mutans Outgrowth and Promoted S. oralis Dominance

**Figure 7:**
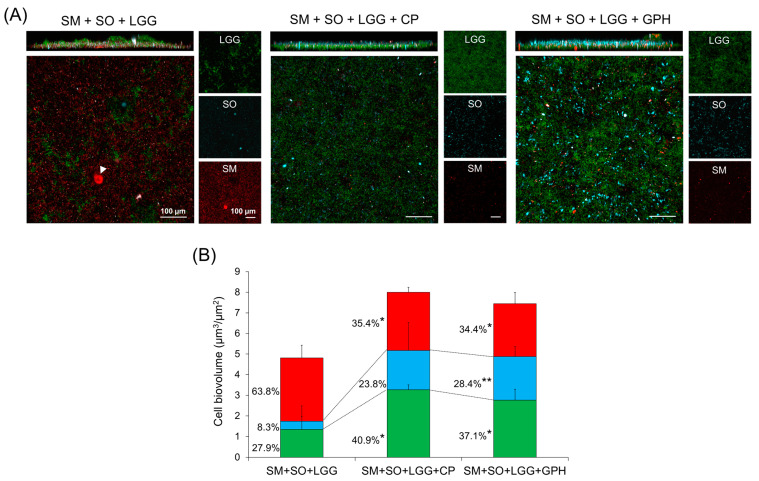
Effects of Gly-Pro-Hyp and CP on a diverse bacteria community in a multi-species biofilm (43 h-old). The mixed bacterial population was inoculated in medium containing 0.1% (*w*/*v*) sucrose and then incubated for 19 h. Then, the initial biofilm was transferred to fresh medium containing 1% sucrose to stimulate a cariogenic challenge from 19 h until 43 h (plus change medium at 28 h). (**A**) Representative confocal images of a multi-species biofilm. Bacterial cells were labeled with species-specific FISH probes. *S. mutans*, *S. oralis,* and *L. rhamnosus* cells are depicted in red, blue, and green, respectively. An arrowhead indicates *S. mutans* microcolony. (**B**) Quantitative analysis of bacteria cell (expressed as biovolume) was performed. Mean ± SD (*n* = 4). A pairwise comparison (vs. SM + SO + LGG) was conducted using student’s *t*-test. * *p* < 0.05, ** *p* < 0.01. SM, *S. mutans*; SO, *S. oralis*; LGG, *L. rhamnosus* GG; CP, collagen peptide; GPH, Gly-Pro-Hyp.

To further confirm the effects of Gly-Pro-Hyp and CP on bacterial diversity in the multi-species community, we selected *S. oralis*, one of the most commonly detected pioneer colonizers in dental biofilms, together with *S. mutans* and *L. rhamnosus*, to form a mixed-species biofilm (42 h-old) via ecological plaque hypothesis. According to our observations, when a biofilm was treated with either Gly-Pro-Hyp or CP, the distribution of *S. mutans* colonies (red) was significantly reduced, while the biovolume of *S. oralis* (blue) had increased gradually. This was especially true in presence of Gly-Pro-Hyp, which showed a 4-fold biovolume ratio increase, but a decrease in the biovolume ratio of *S. mutans* from 63.8% to 34.4% (Figure 7).

This result may be explained by the glycolytic pH drop assay, as Gly-Pro-Hyp and CP decreased the acidogenic and aciduric properties of the *S. mutans* (Table 1), thereby providing a more favorable condition for *S. oralis*, and, in turn, promoting the transition of virulent biofilm (mediated by outgrowth of *S. mutans*) into a less-cariogenic biofilm (*S. oralis* dominated).

## 3. Discussion

Previous microbiome- and imaging-based studies have revealed that a polymicrobial community acts in concert to develop dental plaque biofilms, specifically the supragingival plaque biofilms that are formed on tooth surfaces [20,21,22]. Dysbiosis is mediated by adhesion of cariogenic pathogens to a tooth surface under sucrose-rich and highly acidic milieu. Meanwhile, the cariogenic microbial community is shaped by physical and metabolic interactions with other microorganisms and host factors [6,23].

Depending on disease status and the maturity of the biofilms, the spatial organization of cariogenic pathogens (e.g., EPS-mediated dome-like structure) can be linked to the microbial diagnosis in dental caries along with physicochemical factors associated with localized EPS/acid production and the creation of acidic microenvironments [6,24,25]. Most approaches to the disruption of cariogenic biofilm rely on antimicrobials, though these often are thwarted by the presence of EPS acting as a protective shield, resulting in an enhanced tolerance of EPS-embedded bacteria to antimicrobials. The challenges and limitations of current modalities have generated interest in alternative therapeutic approaches. A proper therapeutic approach against cariogenic biofilms should inhibit the pathogens within the biofilm as it simultaneously disrupts matrix formation [26].

In this study, we introduce a conceptual model in which applying an antagonistic interaction between pathogen and synbiotics may be a feasible means of preventing caries. Using an experimental biofilm model, we confirmed that the presence of *L. rhamnosus* effectively inhibited dome-like biofilm formation by *S. mutans* (reduced dominance of *S. mutans* and EPS scaffolds within the biofilm). Concomitantly, treatment of collagen peptide (CP), together with *L. rhamnosus,* reduced the total biovolume of the biofilm. We also observed that CP is a key modulator of acid production, rendering it capable of altering the microenvironment and maintaining neutral pH in an early-stage biofilm. Thus, a combination of *L. rhamnosus* and CP has greater potential to inhibit cariogenic biofilm formation than a single use of *L. rhamnosus*. Notably, a main tripeptide in collagen, Gly-Pro-Hyp, showed similar activity as CP. Collagen consists of three polypeptide chains (α-chains) that are wrapped around each other to form triple-helical macromolecules, with glycine (Gly) in every third residue [27]. Based on the bioactivity of Gly-Pro-Hyp, the acidogenicity of *S. mutans* was significantly reduced.

How does CP modulate the metabolic traits of *S. mutans*? In this study, *eno*, *ldh*, and *atpD* genes expression was down-regulated, while *aguD* gene expression was up-regulated in *S. mutans* by CP treatment, indicating CP plays a crucial role in metabolic interference via modulating the acidogenic and aciduric properties of *S. mutans* to reduce its cariogenic potential. These findings complement previous laboratory and clinical studies that have demonstrated that exogenous arginine supplementation (as an adjunctive anticaries agent) can modulate cariogenic biofilm formation via the arginine deiminase system in arginolytic bacteria (e.g., *S. gordonii*) by enhancing ammonia production [28,29,30]. Although arginine does not affect *S. mutans* directly, CP can modulate the metabolic traits of *S. mutans*. However, influences of CP on the fitness of commensal bacteria remain unknown, a lacunae that could be addressed through a multi-omics study of the harboring metatranscriptomics and metabolomics in multi-species biofilms. Additionally, it would be interesting to investigate how CP (or Gly-Pro-Hyp) modifies cell metabolism at the cytoplasmic level, which can be done by tracking a fluorescent or radiolabeled peptide on *S. mutans*. This tracking system enables the visualization of a localized peptide in the cytoplasm or on the membrane, or quantification of a metabolized or unmetabolized peptide in a culture medium.

**Figure 8:**
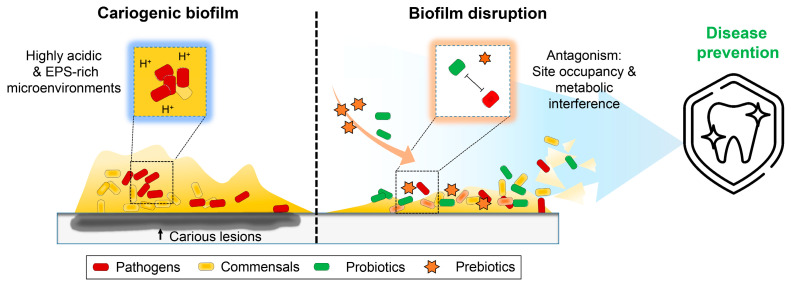
Schematic diagram for synbiotic approach to the disruption of cariogenic biofilm that prevent dental caries.

Since this study is based on well-controlled in vitro assays, further study will be necessary before it can be confirmed whether treatment with probiotics and prebiotics affects the ecology of oral microbiota in vivo, including the proportion and distribution of bacterial species in plaque biofilms. In a future study, we intend to assess the microbial diversity and functional potential of plaque biofilm in a disease or disease-modulating state (dealing with different biofilm developmental stages) as effected by oral-administered oral probiotics and prebiotics. This synbiotic is a potential candidate for an oral health-promoting strategy that operates via inhibiting the virulence potential of *S. mutans* (Figure 8). We anticipate that this will enhance the understanding of the therapeutic potential of synbiotics and their fundamental role in the pathogenesis of severe tooth decay.

## 4. Materials and Methods

### 4.1. Bacterial Strains and Culture Condition

*Streptococcus mutans* UA159 (an established cariogenic dental pathogen and well-characterized EPS producer) were used to generate single- or mixed-species biofilms. *Lactobacillus rhamnosus* GG (LGG^®^, well-characterized and commonly used as a probiotic strain) was provided from Chr. Hansen, Hørsholm, Denmark. This strain maintains genomic and phenotypic stability throughout the industrial freeze-drying process [31]. For inoculum preparation, *S. mutans* and *L. rhamnosus* cells were grown to the mid-exponential phase (optical density at 600 nm (OD_600_) of 0.65 and 0.5, respectively) in ultrafiltered (10-kDa molecular-mass cutoff membrane; Millipore, Danvers, MA, USA) tryptone-yeast extract broth (UFTYE; 2.5% tryptone and 1.5% yeast extract (BD Biosciences, San Jose, CA, USA)) with 1% (*w*/*v*) glucose at 37 °C and 5% CO_2_, using a process described previously [32].

### 4.2. Bacterial Cell Response to Collagen Peptide (CP)

Bacterial cell responses were determined on the agar plate supplemented with CP (Solugel^®^ Prima PP, PB Leiner, Davenport, IA, USA). To assess cell viability of *S. mutans* and *L. rhamnosus* after exposure to CP, a 5 µL-aliquot of serially diluted cell suspensions (with cell densities ranging from 10^3^ to 10^7^ CFU/mL) were spotted on CP containing brain heart infusion (BHI) agar (BD Biosciences) plates. Cell–cell interaction, specifically competition between *L. rhamnosus* and *S. mutans* (10^5^ CFU/mL), involved testing of variable inoculums size (10^5–7^ CFU/mL) of *L. rhamnosus*. To examine the competition, simultaneous point inoculation both strains of which *S. mutans* was spotted on the next to *L. rhamnosus* in BHI agar and incubated for 24 h or 48 h (if the competitive interaction is not observed).

### 4.3. Glycolytic pH Drop Assay

The effect of CP on glycolytic acid production by *S. mutans* planktonic cells was measured, as described elsewhere [33]. Briefly, *S. mutans* or *L. rhamnosus* cells in the planktonic cultures were harvested, washed once with a salt solution (50 mM KCl + 1 mM MgCl_2_, pH = 7), and resuspended in a salt solution. The pH was adjusted to 7.0 with 0.2 M KOH solution. Glucose was then added to obtain a concentration of 1% (*w*/*v*), and pH change was assessed over a period of 90 min using a glass electrode (Orion 3-Star, Thermo Scientific, Waltham, MA, USA). The initial rate of acid production, which provides the best measure of the acid production capacity of the cells, was calculated using pH values.

H-Gly-Pro-Hyp-OH (Bachem, Bubendorf, Switzerland), a common tripeptide unit of collagen, was used for testing. Glycine (Shijiazhuang Shixing Amino Acid Co., Ltd., Shijiazhuang, China), one of most abundant amino acids in CP, was also used to compare the activity of CP. H-Gly-Pro-Hyp-OH, glycine, and CP were used at 5 mg/mL (0.5% (*w*/*v*)).

### 4.4. In Vitro Biofilm Model

Biofilms were formed on hydroxyapatite (HA) discs (surface area, 2.7 ± 0.2 cm^2^) vertically suspended in 24-well plates using a custom-made wire specimen holder. For the in vitro competition model, well-established cariogenic pathogen *S. mutans* and well-characterized probiotic *L. rhamnosus* were grown in UFTYE with 1% glucose at 37 °C and 5% CO_2_. Each of the bacterial suspensions were then mixed to provide an inoculum with a defined microbial population of *S. mutans* (10^5^ CFU/mL) and *L. rhamnosus* (10^7^ CFU/mL). The mixed population was inoculated in 2.8 mL of UFTYE containing 1% (*w*/*v*) sucrose with or without CP (5 mg/mL) and incubated at 37 °C and 5% CO_2_. The culture medium was changed twice daily (at 9 am (19 h) and 6 pm (28 h)) throughout the experimental period (at 19, 23, and 43 h). The EPS were labeled with 1 μM Alexa Fluor 647-dextran conjugate (Molecular Probes, Eugene, OR, USA) [34].

For the three-species biofilm model, pathogen *S. mutans* and commensal *S. oralis* KCTC 13038 (originated from ATCC 35037; this strain obtained from Korean Collection for Type Cultures (KCTC), Korea Research Institute of Bioscience and Biotechnology, Jeongeup, Korea) was used together with *L. rhamnosus*. Each bacterial suspension was mixed to provide an inoculum with *S. mutans* (10^3^), *S. oralis* (10^7^), and *L. rhamnosus* (10^7^). Whole salvia was collected for the preparation of pellicle-coated hydroxyapatite, mimicking the smooth surfaces of a pellicle-coated tooth. Consistent with the ecological plaque hypothesis, as previously established [6,35], the mixed bacterial population was inoculated in UFTYE containing 0.1% (*w*/*v*) sucrose and then incubated for 19 h to form an initially colonized community on the surface. The initial biofilm was then transferred to UFTYE containing 1% sucrose to stimulate a cariogenic challenge at 19 h. The culture medium was changed at 28 h and the biofilm (43 h-old) was subjected to confocal imaging with species-specific labeling [25].

### 4.5. Assessment of Gene Expression via qRT-PCR

RNA was extracted and purified using protocols optimized for biofilms formed in vitro [36]. Briefly, disc sets were incubated in RNALater (Applied Biosystems/Ambion, Austin, TX, USA) and at 43 h the biofilm was removed from the HA discs. The RNAs were purified and DNAse were treated on a column using a Qiagen RNeasy Micro kit (Qiagen, Valencia, CA, USA). The RNAs were then subjected to a second DNaseI treatment with Turbo DNase (Applied Biosystems/Ambion) and purified using the Qiagen RNeasy MinElute Cleanup kit (Qiagen). Then, we performed qRT-PCR to measure the expression profiles of *eno*, *ldh*, *atpD*, and *aguD*. Briefly, cDNAs were synthesized using 0.5 µg of purified RNA and the BioRad iScript cDNA synthesis kit (Bio-Rad Laboratories, Inc., Hercules, CA, USA). The resulting cDNAs were amplified with an Applied Biosystem StepOne system using previously published specific primers: *ldh*, *eno*, *atpD*, *aguD*, and 16S rRNA [36,37,38]. Comparative expression was calculated by normalizing each gene of interest to the 16S rRNA signal [39].

### 4.6. Fluorescence In Situ Hybridization for Bacterial Cell Labeling in Biofilms

3D architecture was analyzed via fluorescence in situ hybridization (FISH), as detailed previously [25]. Bacterial cells were labeled by using species-specific FISH probes: MUT590, 5′-ACTCCAGACTTTCCTGAC-3′ with Cy3 for *S. mutans*; EUB338, 5′-ACAGCCTTTAACTTCAGACTTATCTAA-3′ with FAM for all bacteria, including both *S. mutans* and *L. rhamnosus*. To separate the *L. rhamnosus* from EUB338-labeled cells, the computational subtraction method was applied [25]. For specific labeling of the three species in the multi-species biofilms, MUT590, 5′-ACTCCAGACTTTCCTGAC-3′ with Cy3 for *S. mutans*; Lcai467, 5′-CCGTCACGCCGACAACAG-3′ with FAM for *L. rhamnosus*; MIT588, 5′-ACAGCCTTTAACTTCAGACTTATCTAA-3′ with Cy5 for *S. oralis* [25,40]. The sample in the hybridization buffer (25% formamide, 0.9 M NaCl, 0.01% SDS, 20 mM Tris-HCl, pH 7.2) with the probes was incubated at 46˚C for 4 h. After incubation, the hybridized cells were washed with washing buffer (0.2 M NaCl, 20 mM Tris-HCl (pH 7.5), 5 mM EDTA, 0.01% SDS), and further incubated at 46˚C for 15 min.

### 4.7. Confocal Laser Scanning Microscopy (CLSM)

The 3D biofilm architecture was acquired using C2^+^ confocal microscope (Nikon, Tokyo, Japan) with 20× (0.75 numerical aperture (NA)) or 40× (1.15 NA) objective. The biofilms were sequentially scanned using Diode lasers (488, 568, and 640 nm), and the fluorescence emitted was collected with the PMT detector (490–550 nm for FAM, 565–620 nm for Cy3, and 645–700 nm for Alexa Fluor 647 or Cy5). NIS-Elements software version 5.21 (Nikon) was used to create 3D renderings to visualize the architecture of the biofilms.

Quantitative analysis of cell/EPS biovolume was performed using COMSTAT version 1 (available as free download at http://www.imageanalysis.dk (accessed on 10 January 2022)), written as scripts for MATLAB software (version 9.11, Mathworks, Natick, MA, USA).

### 4.8. Statistical Analysis

Data are presented as mean ± standard deviations (SD). Data were analyzed using analysis of variance (ANOVA) with post hoc Tukey’s HSD test for a multiple comparison or Student’s *t*-test for a pair comparison. Differences between groups were considered statistically significant when *p* < 0.05. Statistical analyses were performed using SPSS version 26.0 software (IBM, Armonk, NY, USA).

## 5. Conclusions

Our data show that the presence of *L. rhamnosus* and CP effectively modulates cariogenic biofilm formation, suggesting that a synbiotic approach may inhibit the virulence potential of *S. mutans*. These findings provide important insights that allow us to further understand the role of synbiotics and potentially develop a combinatorial treatment of probiotics and prebiotics that prevents a prevalent and costly oral disease.

## Figures and Tables

**Table 1:** Profiles of glycolytic pH drop by CP, Gly-Pro-Hyp, and Gly treatments.

Group	pH at 30 min	Delta pH ^1^	[H^+^] Conc. (µM) ^2^	Rate ([H+]/min)	Relative Ratio ^3^
Control	4.73 ± 0.13 ^c,4^	2.26 ± 0.08 ^a^	19.27 ± 5.52 ^a^	0.64 ± 0.18 ^a^	1.00 ± 0.00 ^a^
CP	5.53 ± 0.28 ^ab^	1.18 ± 0.15 ^c^	3.24 ± 2.05 ^bc^	0.11 ± 0.07 ^bc^	0.15 ± 0.06 ^bc^
Gly-Pro-Hyp	5.97 ± 0.37 ^a^	0.75 ± 0.22 ^d^	1.20 ± 1.06 ^c^	0.04 ± 0.04 ^c^	0.05 ± 0.04 ^c^
Gly	5.31 ± 0.20 ^b^	1.44 ± 0.10 ^b^	5.10 ± 2.35 ^b^	0.17 ± 0.08 ^b^	0.25 ± 0.05 ^b^

^1^ Delta pH: initial pH—pH at 30 min after glucose challenge. ^2^ The molar concentration of hydrogen ion in the solution was estimated via the equation: [H+] = 10^-in situ pH^. ^3^ Relative ratio to control (*S. mutans* + *L. rhamnosus* with vehicle). ^4^ Mean ± SD (*n* = 3). Values followed by different superscripts (a–d) are significantly different from each other at *p* < 0.001.

## Data Availability

All gathered data are presented in the main text. Data are available upon request to the authors.
